# Flexible Antenna with Circular/Linear Polarization for Wideband Biomedical Wireless Communication

**DOI:** 10.3390/s23125608

**Published:** 2023-06-15

**Authors:** Mohammed E. Yassin, Khaled F. A. Hussein, Qammer H. Abbasi, Muhammad A. Imran, Shaimaa A. Mohassieb

**Affiliations:** 1 Electronics and Communications Engineering Department, Akhbar Elyom Academy, 6th of October City 12573, Egypt; m.ezzat@akhbaracademy.edu.eg (M.E.Y.); s.mohassieb@akhbaracademy.edu.eg (S.A.M.); 2 Microwave Engineering Department, Electronics Research Institute (ERI), Cairo 11843, Egypt; fkhalid@eri.sci.eg; 3 James Watt School of Engineering, University of Glasgow, Glasgow G12 8QQ, UK; muhammad.imran@glasgow.ac.uk

**Keywords:** circular polarized antenna, compact antenna, flexible antenna, wideband antenna

## Abstract

A wideband low-profile radiating G-shaped strip on a flexible substrate is proposed to operate as biomedical antenna for off-body communication. The antenna is designed to produce circular polarization over the frequency range 5–6 GHz to communicate with WiMAX/WLAN antennas. Furthermore, it is designed to produce linear polarization over the frequency range 6–19 GHz for communication with the on-body biosensor antennas. It is shown that an inverted G-shaped strip produces circular polarization (CP) of the opposite sense to that produced by G-shaped strip over the frequency range 5–6 GHz. The antenna design is explained and its performance is investigated through simulation, as well as experimental measurements. This antenna can be viewed as composed of a semicircular strip terminated with a horizontal extension at its lower end and terminated with a small circular patch through a corner-shaped strip extension at its upper end to form the shape of “G” or inverted “G”. The purpose of the corner-shaped extension and the circular patch termination is to match the antenna impedance to 50 Ω over the entire frequency band (5–19 GHz) and to improve the circular polarization over the frequency band (5–6 GHz). To be fabricated on only one face of the flexible dielectric substrate, the antenna is fed through a co-planar waveguide (CPW). The antenna and the CPW dimensions are optimized to obtain the most optimal performance regarding the impedance matching bandwidth, 3dB Axial Ratio (AR) bandwidth, radiation efficiency, and maximum gain. The results show that the achieved 3dB-AR bandwidth is 18% (5–6 GHz). Thus, the proposed antenna covers the 5 GHz frequency band of the WiMAX/WLAN applications within its 3dB-AR frequency band. Furthermore, the impedance matching bandwidth is 117% (5–19 GHz) which enables low-power communication with the on-body sensors over this wide range of the frequency. The maximum gain and radiation efficiency are 5.37 dBi and 98%, respectively. The overall antenna dimensions are 25 × 27 × 0.13 mm${}^{3}$ and the bandwidth-dimension ratio (BDR) is 1733.

## 1. Introduction

**Figure 1:**
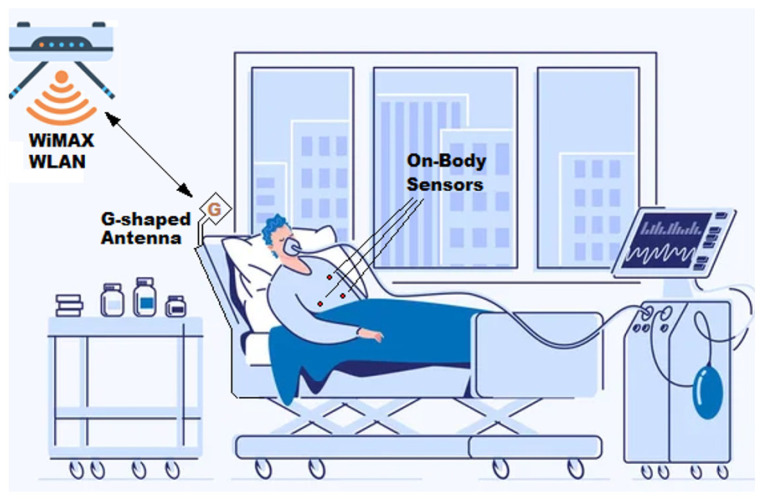
Wireless body area network in intensive care unit where the proposed G-shaped antenna is employed as central off-body antenna for transmitting the biotelemetry data to a nearby WiMAX/WLAN base station antenna.

**Figure 2:**
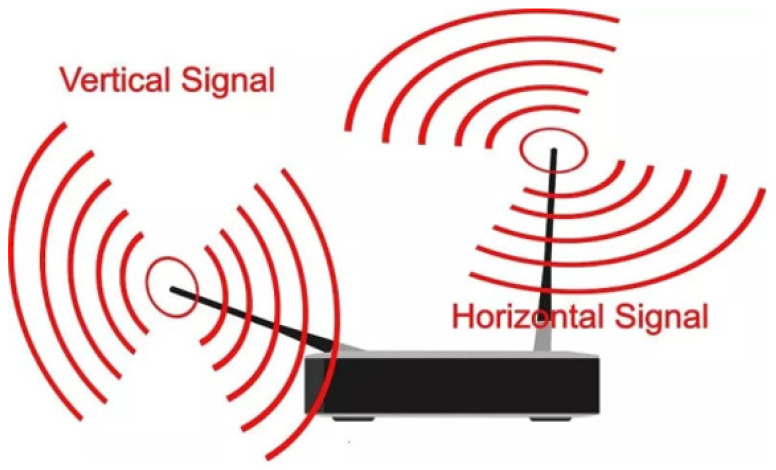
WiMAX/WLAN access point with antennas of arbitrary polarization.

Recently, a lot of research work has been focused on the wideband flexible antennas for biomedical applications [1,2,3,4,5,6,7]. For example, in the application of wireless body area network (WBAN), on-body or implantable biosensors are placed at different positions of the patient body to provide biotelemetry data continuously, at regular time intervals [5,6], or whenever it is required. The biotelemetry data transmitted by the biosensor antennas are collected by a nearby (central) antenna of the WBAN (may be either on-body or off-body antenna). To be available for a wider area network, the central antenna retransmits the collected data to a nearby base station, which is often a WiMAX or Wi-Fi (WLAN) antenna. Figure 1 shows an example of WBAN for a patient in the Intensive Care Unit (ICU) where the G-shaped antenna proposed in the present work is employed as a central off-body antenna to perform the dual function of collecting the biotelemetry data from the biosensors distributed on (or implantable inside) the patient body and then retransmitting them to a WiMAX/WLAN antenna. The minimization of the power consumption is a vital requirement in the biosensor antennas to produce low-power transceivers in biomedical applications, such as the WBAN. To minimize the power required for communication within the nodes of the WBAN, the ultra-wideband (UWB) antennas are preferred to narrowband antennas for their low-power spectral density that is restricted to 41.3 dBm/MHz [8,9]. For this reason, the proposed G-shaped antenna is designed to operate in the “on-body” mode with linear polarization over the wide frequency band (6–19 GHz). In this mode, the central antenna operates with linear polarization to communicate with the biosensor antennas for collecting biotelemetry data. On the other hand, the G-shaped antenna is designed to operate in “off-body” mode for re-transmitting the collected data to the WiMAX/WLAN antenna in the frequency band 5–6 GHz. As shown in Figure 2, the WiMAX/WLAN antenna that may have arbitrary polarization (vertical or horizontal). To reduce the power loss caused by misalignment with the WiMAX/WLAN antenna, the G-shaped antenna is designed to produce circular polarization while operating in the “off-body” mode (5–6 GHz) and linear polarization while operating in the “on-body” mode (6–19 GHz).

The proposed antenna is designed to communicate not only with the biosensors on the body but also with the surrounding medical apparatus attached to the body in a health care system and, also, to communicate with the WLAN access points. To allow better mobility of the patient and the attached medical equipment, this antenna is designed to operate near body and not stuck to the body, as shown in Figure 1. Moreover, this antenna operates in the off-body mode (while communicating with the WLAN) emitting relatively higher level of the power than that emitted in the on-body mode operation. To reduce the specific absorption rate (SAR) in the off-body mode it is preferred not to be placed directly on the patient body. Because of these reasons, the proposed antenna is designed to be placed in a region central to the body sensors, the surrounding medical equipment, and the wireless LAN access point rather than to be integrated into the patient body.

Polarization is a critical and significant feature of antennas in modern wireless communication technology [10]. The orientation of the electric field vector determines the polarization of the antenna, which can be either linear, circular, or elliptical [11]. Antennas with circular polarization (CP) radiate electromagnetic energy in a circular spiral pattern, where the two perpendicular field components are nearly equal in magnitude and in phase quadrature [12]. The enhancement of CP bandwidth is a significant challenge for antenna designers looking to create compact antennas without compromising their performance in wireless communication technologies, such as 5G, Wi-Fi, and WiMax [13].

Lately, different antenna types have been employed to attain circular polarization, including patch antennas, slot antennas, and spiral antennas [14,15,16,17,18,19]. These antennas offer several advantages over traditional linearly polarized antennas, including improved signal quality, lower interference, and increased capacity in wireless communication systems [20,21,22]. In addition to the different antenna types, CP antennas can also be classified based on their feeding networks. Single-feed CP antennas, which utilize a single feed point, are simpler to design and fabricate but have limited bandwidth [23,24,25]. On the other hand, multiple-feed CP antennas, which use multiple feed points, can achieve wider bandwidths but are more complex to design and manufacture [26,27]. Recently, the single-feed co-planar waveguide (CPW) has become the preferred feeding method for wideband CP antennas [28,29,30]. Various types of CPW antennas have been documented to enhance CP performance, including monopole, patch, and slot antennas [31,32,33,34]. These antennas offer wideband circular polarization with high gain and radiation efficiency, making them ideal for use in modern wireless communication technologies.

The flexibility of a circularly polarized (CP) antenna’s substrate can significantly impact its performance [35]. When the antenna is bent or deformed, the polarization purity of the transmitted and received signals can be affected, resulting in a decrease in antenna efficiency and signal quality. However, using a flexible substrate in the design of CP antennas can help mitigate these issues by allowing the antenna to conform to curved or irregular surfaces, reducing the impact of substrate deformation on antenna performance [36,37]. In recent years, there has been a growing interest in designing circularly polarized antennas using flexible substrates to improve their performance under bending conditions. Flexible substrates can conform to curved or irregular surfaces and provide better integration with conformal devices, making them ideal for use in wearable or flexible communication systems [38]. The use of flexible substrates in circularly polarized antennas has been shown to improve radiation performance under bending conditions and reduce the impact of substrate deformation on antenna performance [39,40]. This makes them particularly useful for applications such as smart textiles and Internet of Things (IoT) devices, where antennas need to be compact, low-profile, and able to withstand deformation. As such, research into the design of circularly polarized antennas using flexible substrates has become an active area of investigation in the field of wireless communication technology [41,42,43].

The proposed G-shaped and inverted G-shaped printed strip CP antennas are designed on a low-loss flexible Roger RO3003${}^{TM}$ that exhibits good performance even under bending. The effects of bending the substrate on the antenna parameters were fully investigated by numerical simulations. For enhancing the antenna performance, the antenna is constricted as semicircular wide strip terminated by a small circular patch and fed through a CPW. The geometric dimensions have been optimized for enhanced performance regarding the impedance matching bandwidth, 3dB Axial Ratio (AR) bandwidth, radiation efficiency, and gain.

## 2. Antenna Design

The WiMAX/WLAN antennas may have arbitrary polarization depending on their orientation as shown in Figure 2. To reduce the losses resulting from misalignment of the WBAN central antenna with the WiMAX/WLAN antenna during the off-body mode of operation (5 GHz band), it is proposed that the G-shaped antenna produces circular polarization over the frequency range 5–6 GHz. During the on-body mode of operation (6–19 GHz), it is preferred to operate with linear polarization as it is easier to align the central antenna with the on-body biosensor antennas. The functions assigned to the proposed antenna and the required polarization during the two modes of operation are listed in Table 1.

**Figure 3:**
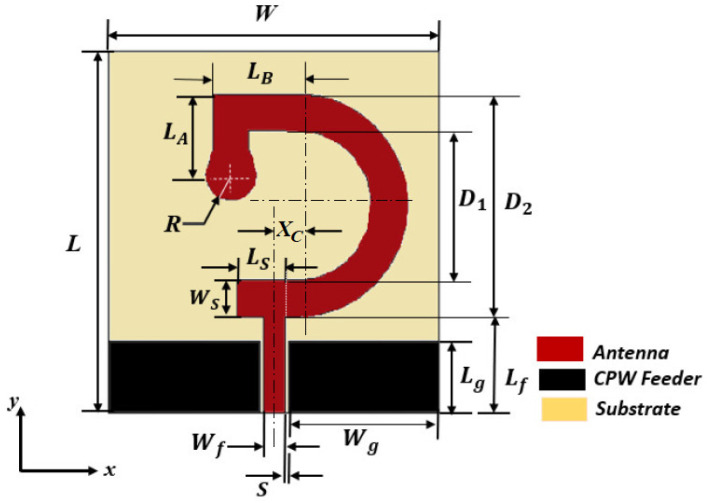
Geometry of the inverted G-shaped strip antenna showing the dimensional parameters.

The G-shaped and inverted G-shaped strip antennas are designed to operate over the frequency band (5–19 GHz). The main issue of the proposed antenna design is to fulfill the requirements of the dual function to produce dual polarization (circular/linear) over a wide frequency band with good impedance matching and high radiation efficiency. The G-shaped radiating strip is a turn-like antenna, i.e., it is similar to a helix or spiral of one turn. Therefore, it can produce both circular and linear polarization by adjusting its dimensions relative to the wavelength. The frequency band 5–6 GHz is actually the unique frequency band that is commonly dedicated for WiMAX and WLAN applications together. Therefore, the frequency band 5–6 GHz is selected for circular polarization. The CST® Studio Suite 3D EM simulator (CST-MWS) is used to design the antenna and evaluate its performance. The geometry of the inverted G-shaped antenna is presented in Figure 3. The inverted-G shaped antenna produces right-hand (CP) in the (+ve z-direction) and left-hand (CP) in the (-ve z-direction) whereas the G-shaped antenna produces left-hand (CP) in the (+ve z-direction) and right-hand (CP) in the (-ve z-direction).

Both the G-shaped and inverted G-shaped antennas can be viewed as composed of semicircular strip connected, at its upper end, to horizontal strip extension of length *L${}_{B}$* followed by vertical strip extension of length *L${}_{A}$* to form a right-angle corner-shaped extension that is terminated by a small circular patch of radius *R*. At its lower end, the semicircular strip is terminated with a horizontal strip extension of length *L${}_{S}$* and width *W${}_{S}$*, as shown in Figure 3. The semicircular strip can be described by the inner and outer diameters *D${}_{1}$* and *D${}_{2}$*, respectively. The antenna is excited through a CPW feeding line. The CPW has a length of *L${}_{g}$*, the width of its central strip is *W${}_{f}$*, and the width of each side slot is *S*. The characteristic impedance of the CPW is obtained based on *W${}_{f}$* and *S* that are calculated using the CPW design equations [44,45]. The length of the CPW central strip extension to connect the antenna to the feed line is *L${}_{f}$*. The antenna and the CPW feeder are printed on a flexible Rogers RO3003${}^{TM}$ substrate which has a dielectric constant $\epsilon_{r}=3,loss\;tangent\;tan\delta =0.001$, and thickness h=0.13 mm. The total size of the printed antenna is *L×W×h*. The best dimensions of the proposed antenna are shown in Table 2.

### 2.1. Evolution of the Antenna Design

**Figure 4:**
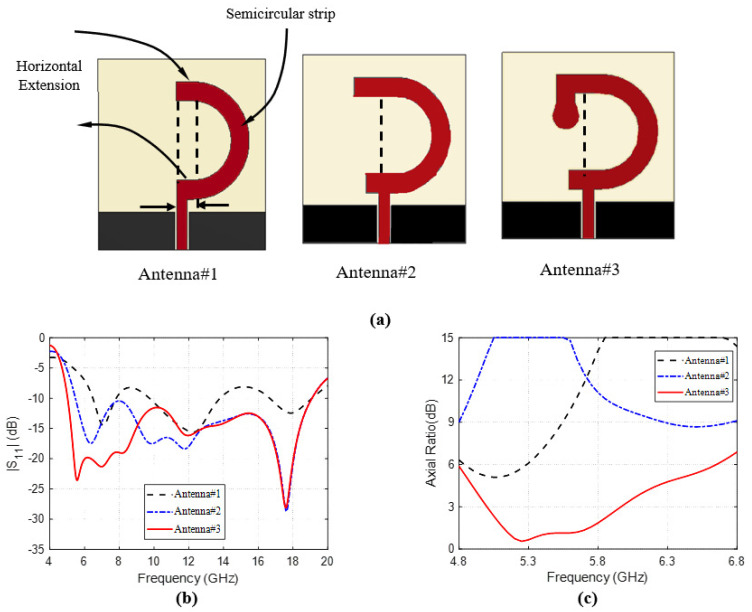
Evolution of the Inverted G-shape CP Antenna Design: (**a**) Evolution steps. (**b**) The reflection coefficient $|S_{11}|$ graph of each design step. (**c**) The AR graph of each design step.

The proposed antenna design has evolved in three main steps as presented in Figure 4a. Other details of the antenna design have been achieved through other minor steps of the design process. However, the major three steps are explained in the present section.

The first step is a trial to produce circular polarization by using a semicircular strip radiator with small horizontal extensions at the end points of the circular arc, as shown in the geometry of Antenna#1. From the curves of $|S_{11}|$ and AR against the frequency Figure 4b,c, it seems that the antenna impedance matching is realized over some frequency bands within the desired wideband and, on the other hand, the AR seems to be improved showing a minimum near 5 GHz. However, neither the desired impedance matching nor the 3dB-AR is achieved.

In the next step of the antenna design, the end points of the radiating strip are further extended to obtain the geometry of Antenna#2. This step of the design results in a great improvement of the impedance matching bandwidth as shown in Figure 4b but, however, the AR is badly affected as shown in Figure 4c. In the third and final major steps of the design process, the strip radiator is extended at its upper end by a vertical extension terminated with a small circular strip as shown in the geometry of Antenna#3. The final design achieves the designed bandwidth of impedance matching, and 3dB-AR as shown in Figure 4b,c.

### 2.2. Investigation of Optimal Dimensions

The G-shaped antenna being proposed consists of five parts, (i) the semicircular strip, (ii) the vertical extension of the central strip of the CPW region, (iii) the horizontal extension at the lower end of the semicircular strip, (iv) the corner extension at the upper end of the semicircular strip, and (v) the small circular patch that terminates the corner extension of the semicircular patch. It is required to obtain the widest impedance matching and 3dB-AR bandwidth and to enhance the radiation efficiency. For this purpose, A thorough investigation of various parameters has been conducted to determine the optimal dimensions of the antenna. The effects of various dimensional parameters on $|S_{11}|$ and AR are numerically investigated in the following subsections.

#### 2.2.1. Effect of the Diameters of the Semicircular Strip

**Figure 5:**
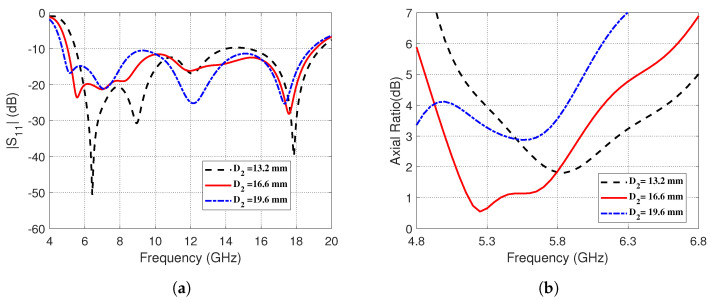
Effect of changing the outer diameter, *D${}_{2}$*, of the semicircular strip on (**a**) The magnitude of the reflection coefficient, $|S_{11}|$, as a function of frequency (4–20 GHz). (**b**) The AR over the frequency range (4.8–6.8 GHz).

The effects of changing the outer and inner diameters, *D${}_{2}$* and *D${}_{1}$*, respectively, are depicted in the present section. The effects of changing the outer diameters *D${}_{2}$* on $|S_{11}|$ and the AR is shown in Figure 5. It is evident in Figure 5a that increasing *D${}_{2}$* leads to a decrease in the lower and higher frequency bands and the entire impedance matching band is shifted towards the left. However, Figure 5b shows that the best performance regarding the 3dB-AR bandwidth is achieved when *D${}_{2}$* = 16.6 mm.

**Figure 6:**
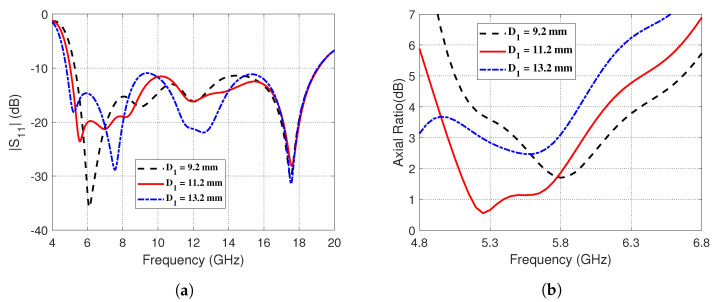
Effect of changing the outer diameter, *D${}_{1}$*, of the semicircular strip on (**a**) the magnitude of the reflection coefficient, $|S_{11}|$, as a function of frequency (4–20 GHz), and (**b**) the AR over the frequency range (4.8–6.8 GHz).

On the other hand, the effects of changing the inner diameter, *D${}_{1}$*, of the semicircular strip on the impedance matching and 3dB-AR bandwidths are presented in Figure 6. Figure 6a shows that increasing *D${}_{1}$* decreases the lower frequency at which the impedance bandwidth matches whereas the higher frequency seems to insensitive to such changes of *D${}_{1}$*. The proposed antenna performs optimally in terms of the 3dB-AR bandwidth when *D${}_{1}$* = 11.2 mm, as illustrated in Figure 6b.

#### 2.2.2. Effects of the Dimensions of the Corner-Shaped Extension of the Curved Strip

**Figure 7:**
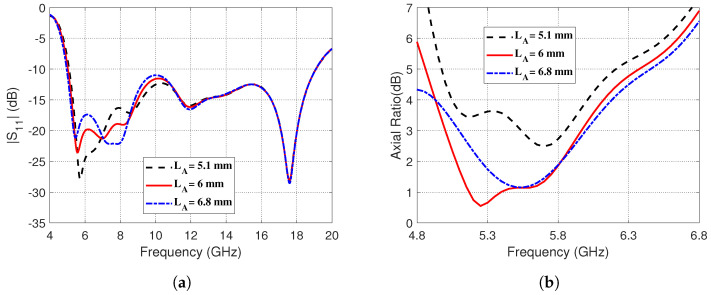
Effect of changing the vertical length, *L${}_{A}$*, of the corner-shaped extension of the curved strip on (**a**) The magnitude of the reflection coefficient, $|S_{11}|$, as a function of frequency (4–20 GHz). (**b**) The AR over the frequency range (4.8–6.8 GHz).

The effects of changing the vertical and horizontal dimensions, *L${}_{A}$* and *L${}_{B}$*, respectively, of the corner-shaped extension of the curved strip on the reflection coefficient magnitude and the AR are investigated in the present section. The effects of changing vertical length, *L${}_{A}$*, on the frequency response of the reflection coefficient magnitude, $|S_{11}|$, and the AR are presented in Figure 7, respectively. It is shown in Figure 7a that the impedance matching frequency band seems to be slightly affected at its lower frequency by changing *L${}_{A}$*. On the other hand, the AR seems to be strongly dependent on *L${}_{A}$*, as shown in Figure 7b. It is clear that setting *L${}_{A}$* = 6 mm gives the best 3dB-AR bandwidth.

**Figure 8:**
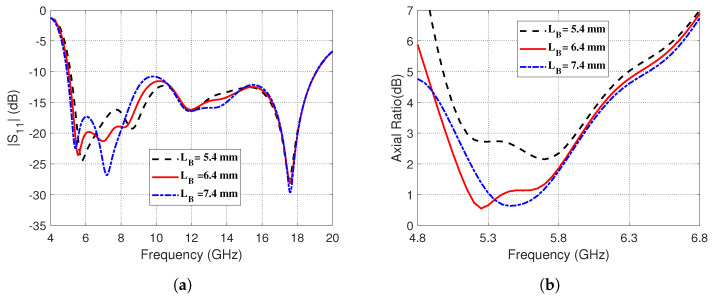
Effect of changing the horizontal length, *L${}_{B}$*, of the corner-shaped extension of the curved strip on (**a**) The magnitude of the reflection coefficient, $|S_{11}|$, as a function of frequency (4–20 GHz). (**b**) The AR over the frequency range (4.8–6.8 GHz).

The effects of changing horizontal length, *L${}_{B}$*, on the frequency response of the reflection coefficient magnitude, $|S_{11}|$, and the AR are presented in Figure 8, respectively. It is shown in Figure 8a that the impedance matching frequency band seems to be slightly affected at its lower frequency by changing *L${}_{B}$*. On the other hand, the AR seems to be strongly dependent on *L${}_{B}$* as shown in Figure 8b. It is clear that setting *L${}_{B}$* = 6.4 mm gives the best 3dB-AR bandwidth.

#### 2.2.3. Influence of the Radius of the Circular Patch Termination

**Figure 9:**
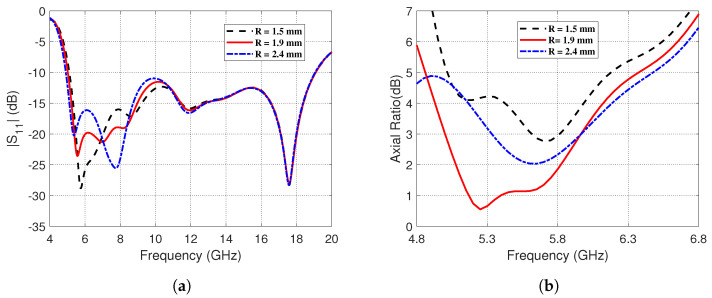
The impact of altering the radius, *R*, of the circular patch termination on (**a**) The reflection coefficient magnitude, $|S_{11}|$, over the frequency range (4–20 GHz). (**b**) The AR over the frequency range (4.8–6.8 GHz).

The impact of altering the radius, *R*, of the small circular patch termination of the G-shaped strip antenna on the frequency dependence of the magnitude of the reflection coefficient, $|S_{11}|$, and the AR are presented in Figure 9. It is shown in Figure 9a that the impedance matching frequency band seems to be slightly affected at its lower frequency by changing *R*. On the other hand, the AR seems to be strongly dependent on *R* as shown in Figure 9b. It is clear that setting *R* = 1.9 mm gives the best 3dB-AR bandwidth.

#### 2.2.4. The Impact of Altering the Length of the CPW Central Strip Extension

**Figure 10:**
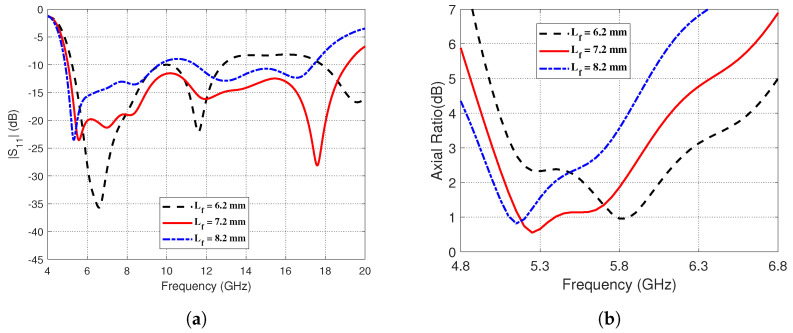
The impact of altering the length, *L${}_{f}$*, of the central strip extension on (**a**) The reflection coefficient magnitude, $|S_{11}|$, over the frequency range (4–20 GHz). (**b**) The AR as a function of frequency (4.8–6.8 GHz).

The impact of altering the length *L${}_{f}$* of the CPW central strip extension on the magnitude of the reflection coefficient, $|S_{11}|$, and the AR over the applicable frequency ranges are shown in Figure 10. Figure 10a shows that the lower frequency of the impedance matching bandwidth slightly decreases with increasing *L${}_{f}$*. However, the widest bandwidth is obtained by setting *L${}_{f}$* = 7.2 mm. This value of *L${}_{f}$* results in a 3dB-AR frequency range covering 5 GHz to 6 GHz as shown in Figure 10b.

#### 2.2.5. Optimum Dimensional Parameters

The parametric sweeps presented in Section 2.2.1, Section 2.2.2, Section 2.2.3 and Section 2.2.4 are examples for the complete investigation of various parameters that has been conducted to determine the optimal values for the geometrical design parameters of the inverted G-shaped antenna. The results of this investigation are presented in Table 2. Furthermore, it should be noted that the dimensions of the CPW region are adjusted to improve the impedance matching where the analytic rules in [44,45] have been used as initial values and then optimized by EM simulation.

## 3. Effect of Bend Stresses on the Inverted G-Shape CP Antenna Characteristics

Owing to its flexible structure, the proposed antenna shall preserve its high performance even while being subjected to bend strains (to some extents) in the different directions.

**Figure 11:**
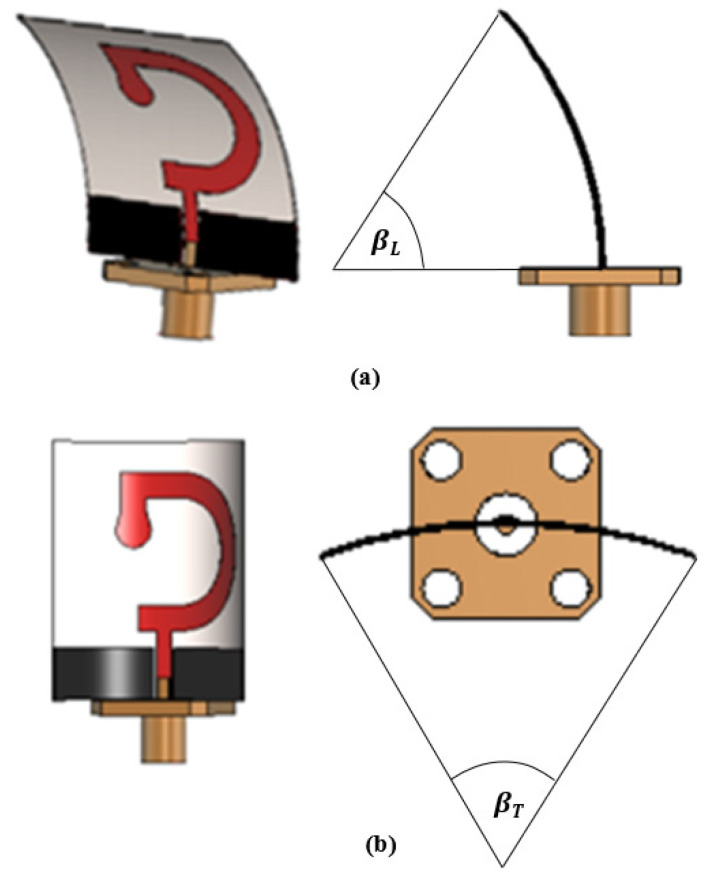
The proposed antenna subjected to bend stresses in (**a**) The longitudinal plane, (**b**) The transverse plane.

For studying the effects of the bend stresses on the characteristics of the proposed antenna, it is subjected to different bend angles (β) and the corresponding frequency dependencies of the reflection coefficient, $|S_{11}|$ and AR are investigated. The bending is applied in one of two perpendicular directions, the bend angle $\beta_{L}$ that is applied in the longitudinal plane parallel to the feeding line as shown in Figure 11a or the bend angle $\beta_{T}$ that is applied in the transverse plane perpendicular to the feed line, as shown in Figure 11b.

### 3.1. Effects of Bending the Antenna in the Longitudinal Plane on the Impedance Matching and Axial Ratio

**Figure 12:**
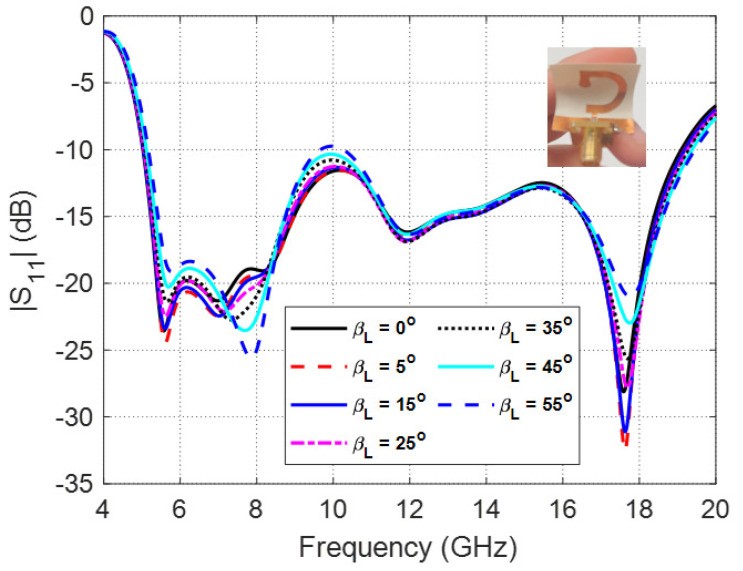
Frequency dependence of the reflection coefficient, $|S_{11}|$, for different values of the longitudinal bend angle, $\beta_{L}$.

The frequency dependence of the reflection coefficient, $|S_{11}|$, for different values of the longitudinal bend angle, $\beta_{L}$ is presented in Figure 12. It is shown that for longitudinal bend angles less than $55^{\circ }$, the dependence of $|S_{11}|$ on the frequency is almost unchanged keeping the impedance matching bandwidth almost the same for $\beta_{L}<55^{\circ }$. However, for $\beta_{L}\geq 55^{\circ }$, the value of $|S_{11}|$ starts to increase above −10 dB leading to decrease the impedance matching bandwidth.

**Figure 13:**
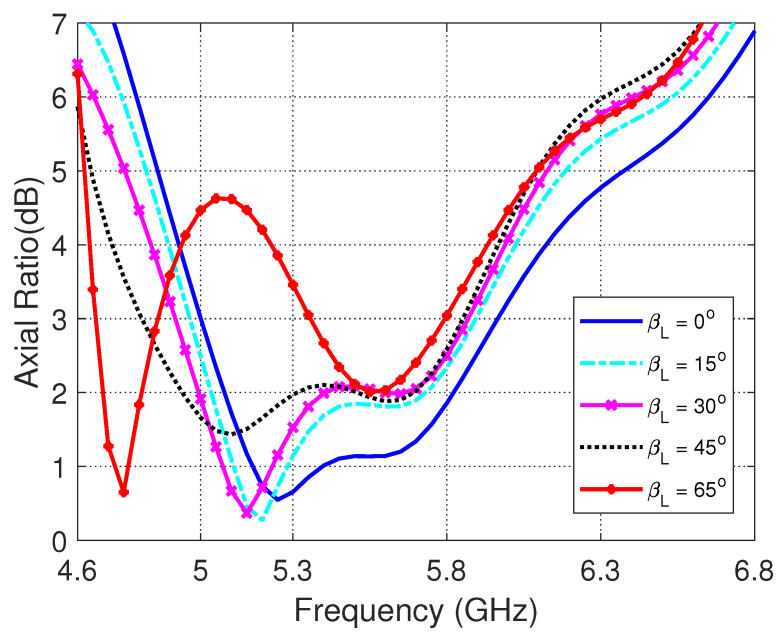
Frequency dependence of the AR for different values of the longitudinal bend angle, $\beta_{L}$.

The frequency dependence of the axial ratio for different values of the longitudinal bend angle, $\beta_{L}$ is presented in Figure 13. It is shown that, with increasing $\beta_{L}$ the frequency band for 3dB-AR is slightly shifted towards the left keeping the 3dB-AR bandwidth almost unchanged keeping the impedance matching bandwidth almost the same for $\beta_{L}<45^{\circ }$. However, for $\beta_{L}\geq 45^{\circ }$, the value of the axial ratio increases above 3 dB leading to lose the circular polarization over a significant part of the frequency band.

### 3.2. Effects of Bending the Antenna in the Transverse Plane on the Impedance Matching and Axial Ratio

**Figure 14:**
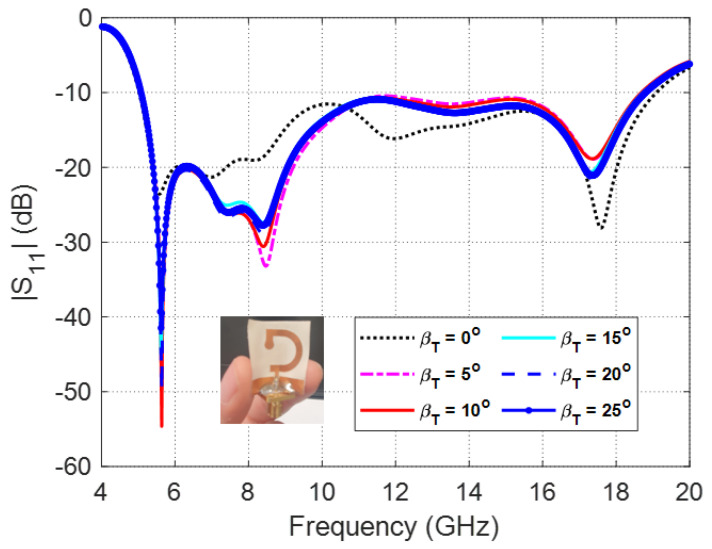
Frequency dependence of the reflection coefficient, $|S_{11}|$, for different values of the transverse bend bend angle, $\beta_{T}$.

The frequency dependence of the reflection coefficient, $|S_{11}|$, for different values of the transverse bend angle, $\beta_{T}$ is presented in Figure 14. It is shown that for $\beta_{T}<25^{\circ }$, the dependence of $|S_{11}|$ on the frequency is slightly affected but, however, the impedance matching bandwidth is almost the same. For $\beta_{T}\geq 25^{\circ }$, the value of $|S_{11}|$ starts to increase above −10 dB leading to decrease the impedance matching bandwidth.

**Figure 15:**
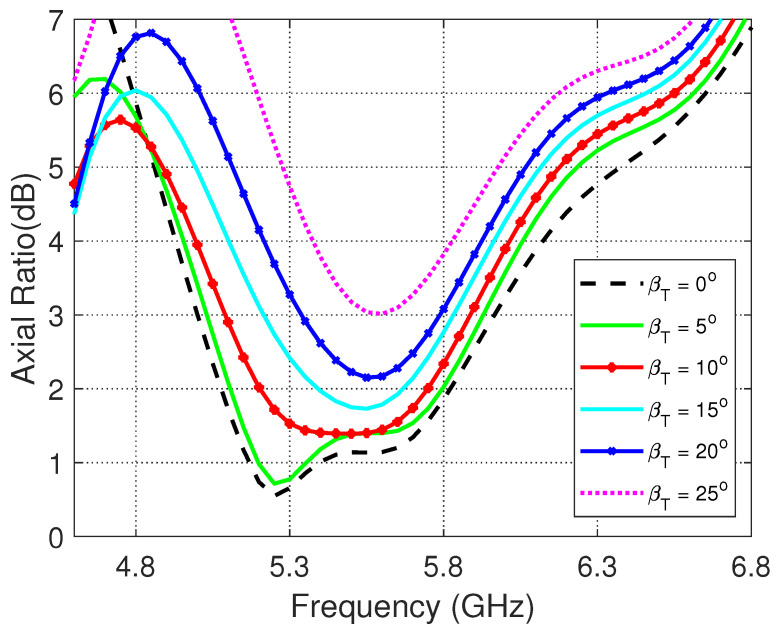
Frequency dependence of the AR for different values of the transverse bend angle, $\beta_{T}$.

The frequency dependence of the axial ratio for different values of the transverse bend angle, $\beta_{T}$ is presented in Figure 15. It is shown that, with increasing $\beta_{T}$ the 3dB-AR bandwidth is continuously decreased as the lower frequency is increased and the higher frequency is decreased. For $\beta_{T}\geq 25^{\circ }$, the minimum value of the axial ratio increases above 3 dB leading to lose the circular polarization over the entire frequency band.

### 3.3. Effect of Bending the Antenna on the Impedance Matching Bandwidth

**Figure 16:**
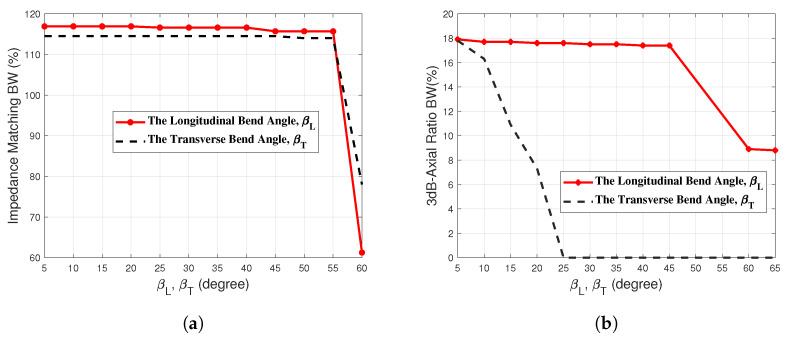
Dependence of the percentage (**a**) impedance matching bandwidth and (**b**) 3dB-AR bandwidth on the bend angles, $\beta_{L}$ and $\beta_{T}$.

The dependence of the percent impedance matching bandwidth on both the longitudinal and transverse bend angles, $\beta_{L}$ and $\beta_{T}$, respectively, is presented in Figure 16a. It is shown that the impedance matching bandwidth is almost independent of the bend angles as long as both $\beta_{L}$ and $\beta_{T}$ are less than $55^{\circ }$. Increasing any of the two bend angles above $55^{\circ }$ causes a dramatic drop of the percent bandwidth of impedance matching.

### 3.4. Effect of Bending the Antenna on the 3dB Axial Ratio Bandwidth

The dependence of the percent bandwidth of 3dB-AR on both the longitudinal and transverse bend angles, $\beta_{L}$ and $\beta_{T}$, respectively, is presented in Figure 16b. It is shown that the impedance matching bandwidth is almost independent of the longitudinal bend angle as long as $\beta_{L}\leq 45^{\circ }$. Increasing $\beta_{L}$ above $45^{\circ }$ causes a dramatic drop of the percent bandwidth 3dB-AR. On the other hand, increasing the transverse bend angle $\beta_{T}$ beyond $5^{\circ }$ leads to a fast decay of the 3dB-AR bandwidth until the circular polarization is completely lost for $\beta_{T}\geq 25^{\circ }$.

Thus, it can be concluded that the proposed antenna preserves the impedance matching bandwidth and the 3dB-AR bandwidth for longitudinal and transverse bend angles less than $55^{\circ }$ (i.e., for $\beta_{L}<55^{\circ }$ and $\beta_{T}<55^{\circ }$). However, to preserve the achieved antenna performance regarding the 3dB-AR bandwidth, it is recommended to keep the longitudinal bend angle less than $45^{\circ }$ and to keep the transverse bend angle less than $5^{\circ }$ (i.e., $\beta_{L}<45^{\circ }$ and $\beta_{T}<5^{\circ }$) otherwise the 3dB-AR bandwidth will be badly affected.

## 4. Mechanism of Circular Polarization

**Figure 17:**
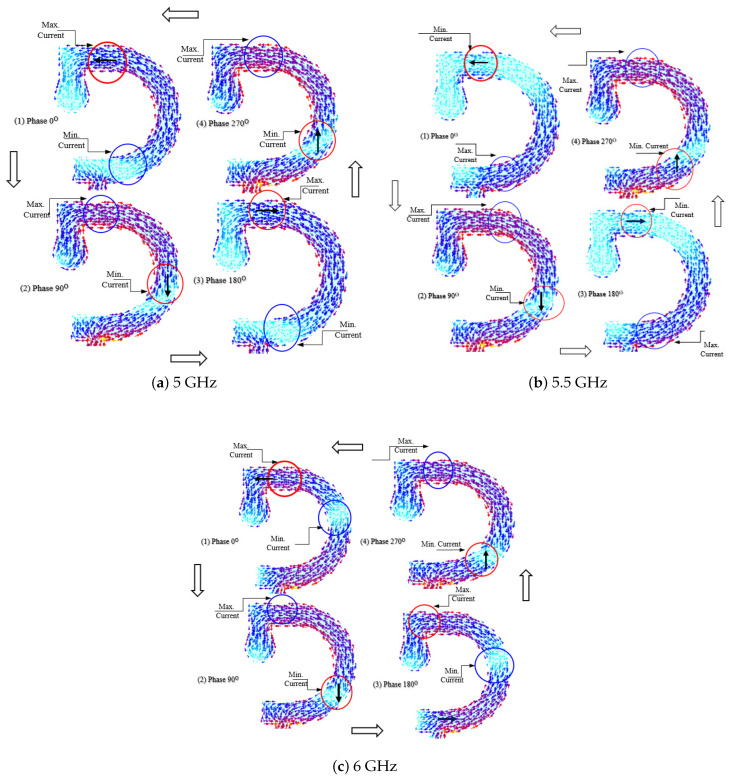
Surface current distributions on the surface of the inverted G-shaped strip antenna at different frequencies range at sequential orthogonal phases (sequential time delays). (**a**) 5 GHz. (**b**) 5.5 GHz, (**c**) 6 GHz.

The way that the proposed inverted G-shaped strip antenna produces circular polarization can be demonstrated by showing the surface current distribution on the semi-circle strip radiator, as depicted in Figure 17. The surface current distributions are presented at sequential orthogonal phases $0^{\circ }$, $90^{\circ }$, $180^{\circ }$, and $270^{\circ }$ which correspond to time delays 0, 1/4T, 1/2T, and 3/4T, respectively, where T is the periodic time at different frequencies, such as 5, 5.5, and 6 GHz.

It is shown that the surface current on the inverted-G-shaped antenna is circulating in the counter clockwise direction, thereby producing RHCP in the +ve z-direction. As the dielectric substrate is very thin relative to the wavelength (thickness =0.002λ) it can be considered transparent to the wave at this frequency. Hence, the amount of power radiated towards the lower half of the space (-ve z-direction) is almost equal to the amount of power radiated towards the upper half of the space (+ve z-direction) but with LHCP.

## 5. Procedure for Measurement of the Radiation Patterns, Gain, and Efficiency

**Figure 18:**
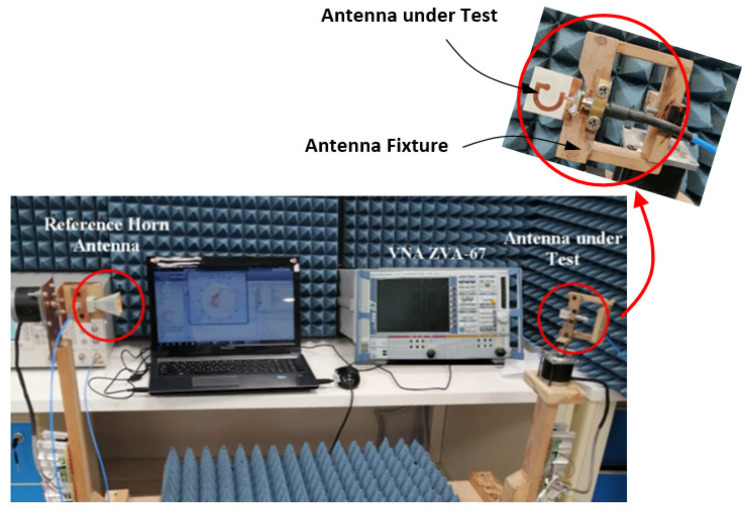
Experimental setup for measurement of the gain, radiation patterns, and antenna efficiency.

This section is concerned with explaining the procedure used to measure the radiation pattern, gain, and efficiency of the proposed antenna. The radiation pattern measurement setup presented in Figure 18, where the antenna under-test is zoomed-in while being mounted on the fixture tool during measurement is established for this purpose. A reference-gain cross-polarized horn antenna is used for gain measurement over the entire frequency band (4–20 GHz). This antenna can be used to separately measure the horizontally and the vertically polarized fields. During measurement, the reference antenna is maintained oriented to the antenna under test (AUT) which is placed on the rotator for complete rotation in the azimuth and elevation planes.

The reference antenna is connected to port 2 of the VNA whereas the AUT is connected to port 1. While rotating the AUT in the desired plane, the readings of the transmission scattering parameter $S_{21}$ are uniformly acquired over the entire frequency band (4–20 GHz) with the preset resolution. A Matlab® program on the laptop plays the role of a central controller and data processor. It controls the rotation of the AUT and the data acquisition of the VNA and stores the $S_{21}$ data during measurement. When the antenna rotation is completed to cover the entire space (0≤θ≤π and 0≤ϕ≤2π) with appropriate angular resolution, where θ and ϕ are spherical angular coordinates related to the coordinate system presented in Figure 3. The stored $S_{21}$ data are processed, the gain is calculated and the radiation patterns at all the frequencies can be drawn. The radiation efficiency over the entire frequency band is also measured. A novel method followed to measure, the radiated field, gain, and radiation efficiency is described as follows.

The effective aperture area of the reference-gain horn antenna (employed as a receiver during measurement) can be expressed as follows

(1) $$A_{REF}=\frac{\lambda^{2}}{4\pi }G_{REF}$$

where λ is the operating wavelength and $G_{REF}$ is the gain of the reference-gain horn antenna.

Let $P_{0}$ be the power output of the wave generator used for transmission during measurement, and $P_{RL}$ be the power returned to the wave generator (the transmitter) due to the impedance mismatch between the AUT and the wave generator. The following expression can be used to evaluate $P_{RL}$.

(2) $$\frac{P_{RL}}{P_{0}}={\left|S_{11}\right|}^{2}$$

The power accepted by the AUT excitation port can be expressed as follows:

(3) $$P_{A}=P_{0}-P_{RL}=P_{0}\left(1-{\left|S_{11}\right|}^{2}\right)$$

### 5.1. Measurement of the Radiation Pattern

Let $P_{R}\left(\theta ,\phi \right)$ be the power received at the reference-gain horn antenna when the rotator directs the AUT at the direction(θ,ϕ). The following expression can be used to evaluate $P_{R}\left(\theta ,\phi \right)$.

(4) $$\frac{P_{R}\left(\theta ,\phi \right)}{P_{0}}=\frac{{\left|S_{21}\left(\theta ,\phi \right)\right|}^{2}}{1-{\left|S_{22}\right|}^{2}}$$

where $S_{21}\left(\theta ,\phi \right)$ is the mutual S-parameter between ports 1 and 2 of the VNA when the rotator directs the AUT towards the direction(θ,ϕ) during measurement and $S_{22}$ is the reflection coefficient measured at port 2 to which the reference-gain antenna is connected during measurement. Thus, the normalized power patterns can be expressed as follows:

(5) $${\hat{P}}_{R}\left(\theta ,\phi \right)=\frac{{\left|S_{21}\left(\theta ,\phi \right)\right|}^{2}}{\max\left({\left|S_{21}\left(\theta ,\phi \right)\right|}^{2}\right)}$$

The far field radiation pattern can be expressed as follows

(6) $$\left|E\left(\theta ,\phi \right)\right|=\sqrt{2\zeta P_{R}\left(\theta ,\phi \right)}$$

where ζ is the intrinsic wave impedance of free space.

Employing (3), the far field radiation pattern:

(7) $$\left|E\left(\theta ,\phi \right)\right|=\sqrt{2\zeta }\frac{\left|S_{21}\left(\theta ,\phi \right)\right|}{\sqrt{1-{\left|S_{22}\right|}^{2}}}$$

The normalized radiation pattern can be evaluated as follows:

(8) $$\hat{E}\left(\theta ,\phi \right)=\frac{\left|S_{21}\left(\theta ,\phi \right)\right|}{\max\left(\left|S_{21}\left(\theta ,\phi \right)\right|\right)}$$

### 5.2. Measurement of the Antenna Gain

The power density at the reference-gain antenna when the AUT is directed at (θ,ϕ) can be expressed as follows:

(9) $$\rho \left(\theta ,\phi \right)=\frac{P_{A}G_{A}\left(\theta ,\phi \right)}{4\pi D^{2}}$$

where $G_{A}\left(\theta ,\phi \right)$ is the gain of the AUT in the direction (θ,ϕ), D is the distance between the transmitting and receiving antennas during measurement. The power received at the reference-gain antenna when the AUT is directed at (θ,ϕ) can be expressed as follows.

(10) $$P_{R}\left(\theta ,\phi \right)=\rho \left(\theta ,\phi \right)\frac{A_{REF}}{\eta_{REF}}$$

where $\eta_{REF}$ is the radiation efficiency of the reference-gain antenna. It is used in the denominator of the right-hand side of (10) to compensate for the losses of the reference-gain antenna other than the return loss.

Substituting from (9) into (10), the power at the receiving antenna can be expressed as follows:

(11) $$P_{R}\left(\theta ,\phi \right)=\frac{P_{A}G_{A}\left(\theta ,\phi \right)}{4\pi D^{2}}\frac{A_{REF}}{\eta_{REF}}$$

Making use of (1), (3), and (11), the power received at the reference-gain antenna can be expressed as follows:

(12) $$P_{R}\left(\theta ,\phi \right)=P_{0}\left(1-{\left|S_{11}\right|}^{2}\right)G_{A}\left(\theta ,\phi \right)\frac{\lambda^{2}}{{\left(4\pi D\right)}^{2}}\frac{G_{REF}}{\eta_{REF}}$$

Thus, the AUT antenna gain, $G_{A}\left(\theta ,\phi \right)$, can be expressed as follows:

(13) $$G_{A}\left(\theta ,\phi \right)=\frac{P_{R}\left(\theta ,\phi \right)}{P_{0}\left(1-{\left|S_{11}\right|}^{2}\right)}\frac{{\left(4\pi D\right)}^{2}}{\lambda^{2}}\frac{\eta_{REF}}{G_{REF}}$$

Substituting from (4) into (13), the following expression is obtained.

(14) $$G_{A}\left(\theta ,\phi \right)=\frac{{\left|S_{21}\left(\theta ,\phi \right)\right|}^{2}}{\left(1-{\left|S_{11}\right|}^{2}\right)\left(1-{\left|S_{22}\right|}^{2}\right)}\frac{{\left(4\pi D\right)}^{2}}{\lambda^{2}}\frac{\eta_{REF}}{G_{REF}}$$

Note that $S_{11}$, $S_{22}$, and $S_{21}\left(\theta ,\phi \right)$ are measured by the VNA and all the other parameters on the right-hand side of (14) are known before carrying out the measurement procedure. Hence, the expression (14) can be used for measuring the gain of the AUT.

The realized antenna gain of the AUT can be expressed as follows:

(15) $$G_{R}\left(\theta ,\phi \right)=\left(1-{\left|S_{11}\right|}^{2}\right)G_{A}\left(\theta ,\phi \right)=\frac{{\left|S_{21}\left(\theta ,\phi \right)\right|}^{2}}{\left(1-{\left|S_{22}\right|}^{2}\right)}\frac{{\left(4\pi D\right)}^{2}}{\lambda^{2}}\frac{\eta_{REF}}{G_{REF}}$$

### 5.3. Measurement of the Antenna Efficiency

The power received by the reference-gain horn antenna can be obtained by calculating the following double integral

(16) $$P_{Rad}=\int_{0}^{2\pi }\int_{0}^{\pi }\rho \left(\theta ,\phi \right)D^{2}\sin\theta d\theta d\phi$$

where ρ(θ,ϕ) is the power density at the location of the reference-horn antenna (the receiver) when the AUT is directed at (θ,ϕ) by the rotator.

The total power radiated by the AUT can be calculated using the following double integral.

(17) $$P_{Rad}=\frac{4\pi D^{2}}{\lambda^{2}}\frac{\eta_{REF}}{G_{REF}}\int_{0}^{2\pi }\int_{0}^{\pi }P_{R}\left(\theta ,\phi \right)\sin\theta d\theta d\phi$$

#### 5.3.1. Total Antenna Efficiency

Dividing both sides of (17) by $P_{0}$, the following expression is obtained for the total efficiency of the AUT.

(18) $$\eta_{\mathrm{Total}\;}=\frac{P_{Rad}}{P_{0}}=\frac{4\pi D^{2}}{\lambda^{2}}\frac{\eta_{REF}}{G_{REF}}\int_{0}^{2\pi }\int_{0}^{\pi }\frac{P_{R}\left(\theta ,\phi \right)}{P_{0}}\sin\theta d\theta d\phi$$

Substituting from (4) into (18), the following expression is obtained.

(19) $$\eta_{\mathrm{Total}\;}=\frac{P_{Rad}}{P_{0}}=\frac{1}{1-{\left|S_{22}\right|}^{2}}\frac{4\pi D^{2}}{\lambda^{2}}\frac{\eta_{REF}}{G_{REF}}\int_{0}^{2\pi }\int_{0}^{\pi }{\left|S_{21}\left(\theta ,\phi \right)\right|}^{2}\sin\theta d\theta d\phi$$

#### 5.3.2. Antenna Radiation Efficiency

The radiation efficiency of the AUT can be expressed as follows.

(20) $$\eta_{Rad}=\frac{P_{Rad}}{P_{0}-P_{RL}}$$

The expression (20) can be reformulated as follows.

(21) $$\eta_{\mathrm{Rad}\;}=\frac{P_{Rad}}{P_{0}}{\left(1-\frac{P_{RL}}{P_{0}}\right)}^{-1}$$

Making use of (2) and (19), the expression (21) can be reformulated so that radiation efficiency can be calculated as follows.

(22) $$\eta_{Rad}=\frac{1}{\left(1-{\left|S_{22}\right|}^{2}\right)\left(1-{\left|S_{11}\right|}^{2}\right)}\frac{4\pi D^{2}}{\lambda^{2}}\frac{\eta_{REF}}{G_{REF}}\int_{0}^{2\pi }\int_{0}^{\pi }{\left|S_{21}\left(\theta ,\phi \right)\right|}^{2}\sin\theta d\theta d\phi$$

## 6. Experimental Results and Discussions

In this section, the antenna fabrication is described, and the experimental assessment of the antenna performance are presented and compared to the simulation results.

### 6.1. Fabrication and Measurements of Reflection Coefficient

**Figure 19:**
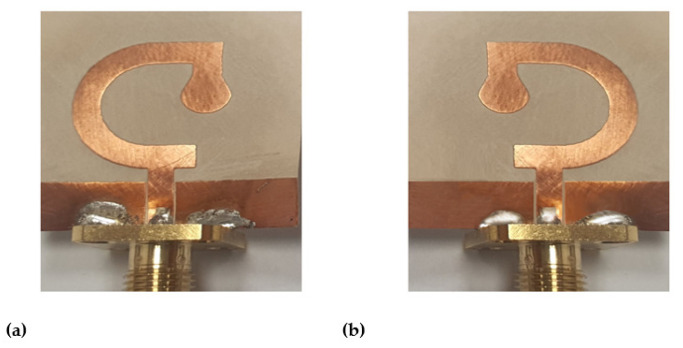
A photograph of the fabricated antennas. (**a**) G-shaped strip antenna. (**b**) Inverted G-shaped strip antenna.

For experimental investigations, both the G-shaped and inverted G-shaped antennas are fabricated. The antenna prototypes are presented in Figure 19. Each of them is subjected to experimental measurement of the input impedance and radiation characteristics.

**Figure 20:**
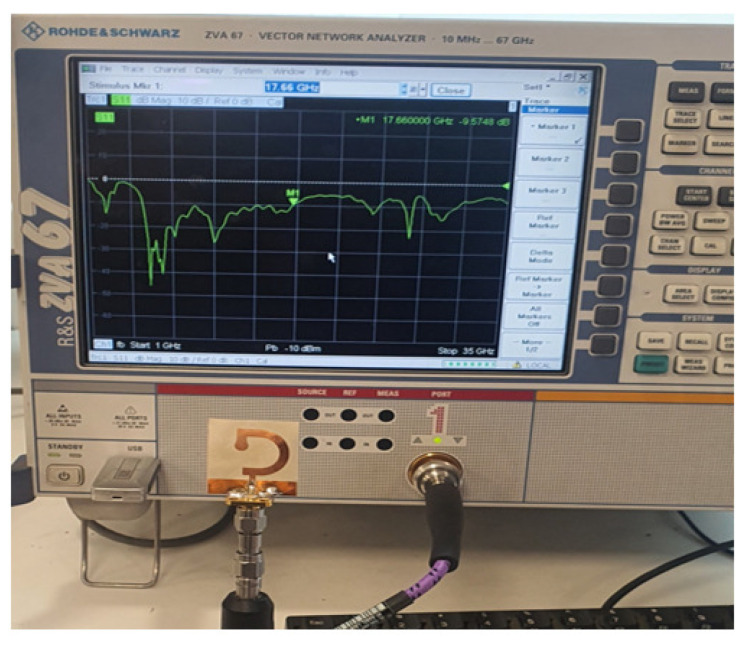
The fabricated prototype of the inverted G-strip antenna is connected to the VNA to measure the reflection coefficient magnitude, $|S_{11}|$, at the antenna feeding port.

**Figure 21:**
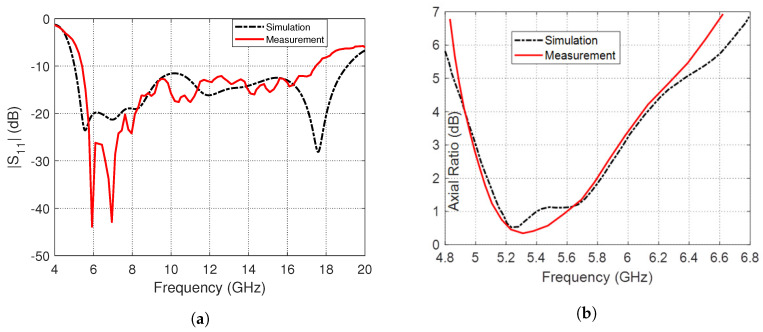
Frequency response as obtained by simulation compared to that obtained by measurement for the inverted G-shaped strip antenna (**a**) The $|S_{11}|$. (**b**) The AR.

To measure the reflection coefficient magnitude, $|S_{11}|$, the antenna is connected to Rohde and Schwarz vector network analyzer (VNA) model ZVA 67, as shown in Figure 20. The frequency dependence of $|S_{11}|$ is presented in Figure 21a where the experimental and simulation results come in good agreement with each other, indicating that the impedance bandwidth is about 14 GHz (5–19 GHz). The change in the AR over the frequency range of (4.8–6.8 GHz) is shown in Figure 21b. The measurement results are consistent with the simulation results, indicating that the 3dB-AR bandwidth is around 1 GHz (5–6 GHz), i.e., 18% percentage fractional bandwidth.

The antenna is designed to produce circular polarization over the frequency range 5–6 GHz (for the 5GHz band of the WLAN/WiMAX applications) with left-hand sense in the directions of one half-space and right-hand polarization in the opposite directions of the other half-space. On the other hand, it is designed to produce linear polarization over the frequency range 6–19 GHz. This makes the proposed antenna more able to operate in the applications requiring wideband with varying polarization characteristics.

The frequency bands allocated for the WiMAX and WLAN applications are listed in the in Table 3. The proposed antenna is well-suited to operate in the 5 GHz band allocated for both applications [46].

### 6.2. Gain and Radiation Efficiency

**Figure 22:**
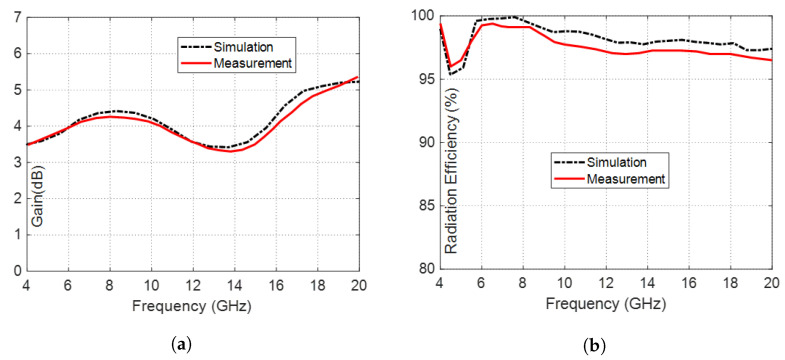
The inverted G-shaped antenna exhibits a dependency on frequency, as obtained by simulation and measurement over the frequency range (4–20 GHz) for (**a**) The Gain. (**b**) The radiation efficiency.

The dependence of the maximum gain generated by the inverted G-shaped antenna on the frequency is shown in Figure 22a. Both simulation and experimental results indicate that the maximum gain varies between 3.5 dBi and 5.37 dBi over the frequency band of impedance matching (5–19 GHz) and between 3.5 dBi and 3.8 dBi over the 3dB-AR frequency band (5–6 GHz). This means that the maximum gain produced by the proposed G-shaped and inverted G-shaped antennas is relatively stable over the frequency band of concern.

The results of the simulation and experimental testing of the radiation efficiency of the inverted G-shaped antenna, which were conducted over the frequency range of (4–20 GHz), are presented in Figure 22b and demonstrate good agreement. Owing to the coplanar structure and the high quality and small thickness of the substrate material, the radiation efficiency is maintained above 98% over almost the entire frequency band of operation (5–19 GHz).

### 6.3. Radiation Patterns

#### 6.3.1. Circularly Polarized Radiated Fields

**Figure 23:**
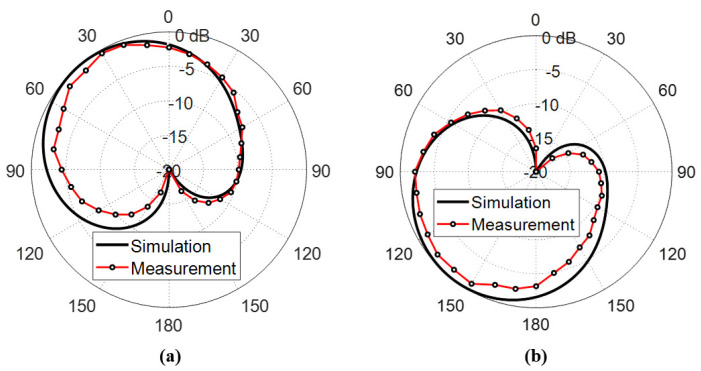
Radiation patterns of (**a**) RHCP and (**b**) LHCP fields produced by the inverted G-shaped antenna in the plane $\phi =0^{\circ }$ at the 5 GHz.

**Figure 24:**
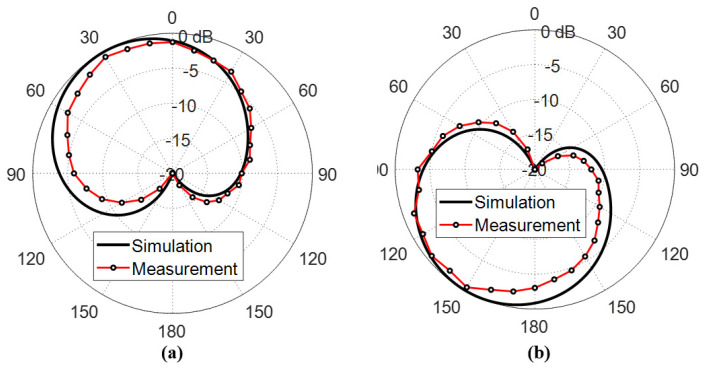
Radiation patterns of (**a**) RHCP and (**b**) LHCP fields produced by the inverted G-shaped antenna in the plane $\phi =0^{\circ }$ at the 5.5 GHz.

**Figure 25:**
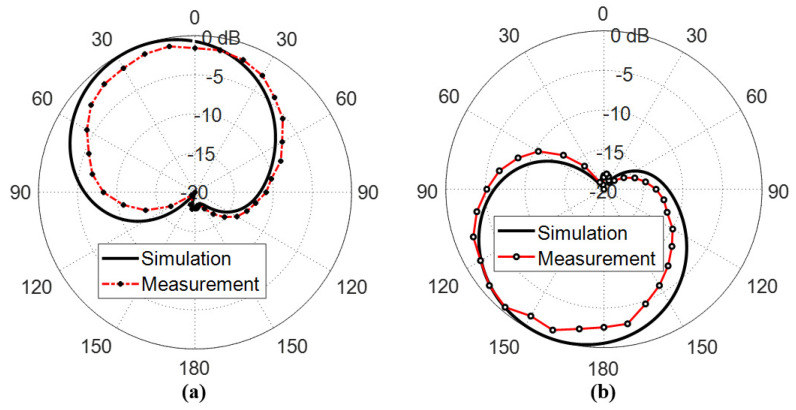
Radiation patterns of (**a**) RHCP and (**b**) LHCP fields produced by the inverted G-shaped antenna in the plane $\phi =0^{\circ }$ at the 6 GHz.

The measured far field radiation patterns of the circularly polarized components E-left and E-right generated by the inverted G-shaped antenna have been added and compared to those obtained by simulation in the plane $\phi =0^{\circ }$ at 5, 5.5, and 6 GHz, as shown in Figure 23, Figure 24, and Figure 25, respectively.

It is shown that the radiated field of the inverted G-shaped antenna is dominated by RHCP component in upper half-space (+ve z-direction) and LHCP component in the lower half of the space (-ve z-direction).

#### 6.3.2. Linearly Polarized Radiated Fields

**Figure 26:**
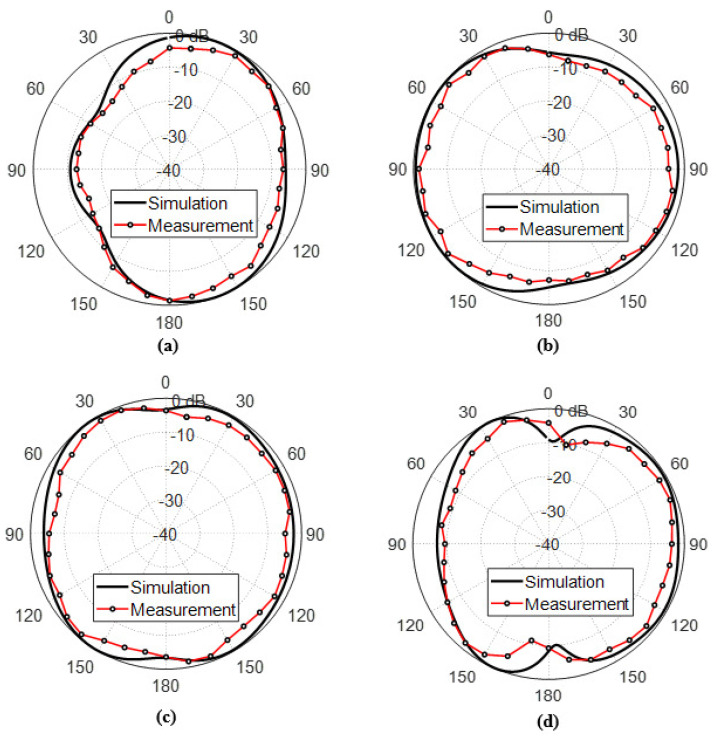
Radiation patterns of the total electric field produced by the inverted G-shaped antenna (Linearly Polarized) in the plane $\phi =0^{\circ }$ (**a**) 9 GHz, (**b**) 12 GHz, (**c**) 15 GHz, and (**d**) 18 GHz.

The measured far field radiation patterns of the total electric field (Linearly Polarized) produced by the inverted G-shaped in the plane $\phi =0^{\circ }$ at 9, 12, 15, and 18 GHz, respectively, as shown in Figure 26. The measured and the simulated radiation patterns of the total radiated field at multiple frequencies over the frequency range 6–19 GHz show good agreement.

### 6.4. Summary of Comparative Performance

To demonstrate the contribution of the present work in the context of the published work with similar interest, some performance measures of the proposed G-shaped antenna are provided in Table 4 in comparison to those of other CP antenna designs proposed in some recent publications.

Table 4 includes designs for rigid and flexible antennas. The flexibility of the antenna is required especially for the medical applications where a central Wireless Body Area Network (WBAN) antenna should be conformal to the surface on which it is mounted. Such a type of antenna has dual vital functions of communication with the on-body biosensor antennas and the off-body LAN antennas. In the off-body mode this antenna communicates with the WiMAX/WLAN antenna to send the data collected form the body sensors to the LAN so that the doctors can take care of their patients remotely.

By comparison with the other antennas listed in Table 4, it becomes clear that the antenna proposed in the present work is the unique antenna that is flexible, operating with circular polarization over one of the frequency bands of the WiMAX/WLAN applications (4.9–6.0 GHz; see Table 3), and operating with linear polarization over the very wide band (6–19 GHz). The circular polarization is necessary while communicating with the LAN antenna as the patient movements result in misalignment between the attached central antenna and the WiMAX/WLAN antenna, which would degrade the signal level if the antenna were linearly polarized.

On the other hand, the proposed antenna can use the remaining part of the operational band (6–19 GHz) for high data rate and wideband communication with the on-body (and in-body) biosensor antennas attached to (or implantable in) the patient body through linear polarization. During this mode of operation, the alignment between the proposed antenna and the biosensor antennas can be easily maintained. Regarding the above requirements (flexibility, circular polarization in the band 4.9–6.0 GHz, and linear polarization over the wideband 6–19 GHz) can be uniquely satisfied by the antenna proposed in the present work when compared to the other antennas listed in Table 4.

## 7. Conclusions

A wideband low-profile G-shaped strip antenna on a flexible substrate has been proposed for biomedical for the application of WBAN to work as off-body communication. The antenna produces circular polarization over the frequency band 5–6 GHz to communicate with WiMAX/WLAN antennas and to produce linear polarization over the frequency band 6–19 GHz for communication with the biosensor antennas. It has been shown that an inverted G-shaped strip produces CP of the opposite sense to that produced by G-shaped strip over the frequency range 5–6 GHz. The antenna structure is composed of a semicircular strip terminated with a horizontal extension at its lower end and terminated with a small circular patch through a corner-shaped strip extension at its upper end to form the shape of “G” or inverted “G”. The corner-shaped extension and the circular patch termination has been added to the antenna geometry for impedance matching over the entire frequency band (5–19 GHz) and to improve the circular polarization over the frequency band (5–6 GHz). The antenna has been fed through a CPW to allow the fabrication on only one face of the flexible dielectric substrate. The dimensions of the antenna as well as the CPW have been optimized to obtain the best performance regarding the impedance matching bandwidth, 3dB-AR bandwidth, radiation efficiency, and maximum gain. The results have shown that the achieved 3dB-AR bandwidth is 18% (5–6 GHz) to cover the 5 GHz frequency band of the WiMAX/WLAN applications within its 3dB-AR frequency band. Furthermore, the impedance matching bandwidth has been shown to reach 117% (5–19 GHz) which enables low-power communication with the biosensors over this wide range of the frequency. It has been shown that the maximum gain and radiation efficiency are 5.37 dBi and 98%, respectively. The antenna dimensions are 25 × 27 × 0.13 mm${}^{3}$ and the BDR is 1733.

## Figures and Tables

**Table 1:** Operations assigned to the G-shaped strip antenna proposed for WBAN in ICU.

Mode of Operation	Function of the Antenna	Frequency Range	Polarization
On-body	Communicate with the biosensor	6–19 GHz	Linear
	antennas for biotelemetry		
Off-body	Retransmit biotelemetry to	5–6 GHz	Circular
	WiMAX/WLAN antennas		

**Table 2:** Optimum dimensions of the proposed CP antenna.

Parameter	*W*	*L*	*D* ${}_{1}$	*D* ${}_{2}$	*R*	*L* ${}_{S}$	*h*	*W* ${}_{S}$
**Value (mm)**	25	27	11.2	16.6	1.9	4.6	0.13	2.7
**Parameter**	***L* ${}_{A}$ **	***L* ${}_{B}$ **	***W* ${}_{g}$ **	***L* ${}_{g}$ **	***L* ${}_{f}$ **	***W* ${}_{f}$ **	***S***	***X* ${}_{C}$ **
**Value (mm)**	6	6.4	11.4	5.4	7.2	1.6	0.3	1.8

**Table 3:** The WiMAX and WLAN frequency bands application.

Name of the Allocated Band	WiMAX	WLAN
700 MHz band	470–862 MHz	–
1.4 GHz band	1.390–1.435 GHz	–
2.3 GHz band	2.300–2.400 GHz	–
2.4 GHz band	2.498–2.800 GHz	–
2.5 GHz band	–	2.400–2.484
3.5 GHz band	3.300–3.800 GHz	–
3.6 GHz band	–	3.675–3.890
5 GHz band	4.900–5.980 GHz	4.915–5.925

**Table 4:** A comparison of the performance measures of the G-shaped antenna with those of other antenna designs that have been recently published.

Work	Dimensions (mm×mm)	Frequency Band (GHz)	% BW for Impedance Matching	Radiation Efficiency	Gain (dBi)	% BW for 3dB-AR	Substrate
[1]	35×35	5.6–6.1	6.6%	NA	7.2	3.8%	Flexible
[4]	65×65	1.4–1.8	25.4%	NA	−1.25	30.1%	Flexible
[21]	20×20	5.2–6.8	13.7%	NA	2.94	6.8%	Flexible
[30]	13×13.4	4.8–16	118%	NA	3.33	55.8%	Rigid FR-4
[47]	29×46.5	2.1–3.6	60%	97%	4.5	13%	Flexible
[48]	32×38	5.5–7	24%	NA	NA	16.6%	Flexible
[49]	3×5	3.4–7	69%	85%	6.5	21.8%	Rigid FR-4
[50]	50×50	1.8–2.4	37.4%	NA	3.95	15.5%	Rigid FR-4
[51]	31×38	3.8–4.5	17.53%	91%	3.1	10.47%	Flexible
[52]	39×39	2.22–9.92	126.5%	80%	3.98	73.3%	Rigid FR-4
Present	25×27	4.9–19	117%	98%	5.37	18%	Flexible

## Data Availability

Not applicable.

## Abbreviations

The following abbreviations are used in this manuscript:

CP	Circularly Polarized
CPW	Co-planar Waveguide
WBAN	Wireless Body Area Network
UWB	Ultra-wideband
RHCP	Right-hand CP
LHCP	Left-hand CP
AR	Axial Ratio
BDR	Bandwidth-Dimension Ratio
VNA	Vector Network Analyzer
